# Distinct Phenotypes Induced by Three Degrees of Transverse Aortic Constriction **in Mice**

**DOI:** 10.1038/s41598-019-42209-7

**Published:** 2019-04-10

**Authors:** Daniel A. Richards, Mark J. Aronovitz, Timothy D. Calamaras, Kelly Tam, Gregory L. Martin, Peiwen Liu, Heather K. Bowditch, Phyllis Zhang, Gordon S. Huggins, Robert M. Blanton

**Affiliations:** 1 Molecular Cardiology Research Institute, Tufts Medical Center, 800 Washington Street, Boston, Massachusetts 02111 USA; 2 Sackler School of Graduate Biomedical Sciences, Tufts University, 145 Harrison Avenue, Boston, MA 02111 United States

## Abstract

Transverse aortic constriction (TAC) is a well-established model of pressure overload-induced cardiac hypertrophy and failure in mice. The degree of constriction “tightness” dictates the TAC severity and is determined by the gauge (G) of needle used. Though many reports use the TAC model, few studies have directly compared the range of resulting phenotypes. In this study adult male mice were randomized to receive TAC surgery with varying degrees of tightness: mild (25G), moderate (26G) or severe (27G) for 4 weeks, alongside sham-operated controls. Weekly echocardiography and terminal haemodynamic measurements determined cardiac remodelling and function. All TAC models induced significant, severity-dependent left ventricular hypertrophy and diastolic dysfunction compared to sham mice. Mice subjected to 26G TAC additionally exhibited mild systolic dysfunction and cardiac fibrosis, whereas mice in the 27G TAC group had more severe systolic and diastolic dysfunction, severe cardiac fibrosis, and were more likely to display features of heart failure, such as elevated plasma BNP. We also observed renal atrophy in 27G TAC mice, in the absence of renal structural, functional or gene expression changes. 25G, 26G and 27G TAC produced different responses in terms of cardiac structure and function. These distinct phenotypes may be useful in different preclinical settings.

## Introduction

Heart failure remains a leading cause of morbidity and mortality worldwide^1^. In humans, heart failure represents a very heterogeneous syndrome, encompassing both varying degrees of cardiac remodelling and dysfunction, as well as a spectrum of clinical phenotypes ranging from asymptomatic to the decompensated state, which typically involves elevated end diastolic pressure and/or reduced cardiac output^2–4^. Preclinical studies continue to rely heavily on mouse models of heart failure, making the accuracy and validity of these models imperative both for mechanistic studies and for drug development^5–8^.

Transverse aortic constriction (TAC) is one of the most common surgical models of pressure overload-induced cardiac hypertrophy and heart failure^9,10^. In the TAC model a permanent constriction is placed around the transverse aorta, limiting left ventricular (LV) outflow and thereby creating pressure overload in the LV. TAC can produce profound adverse cardiac remodelling such as hypertrophy and fibrosis, as well as systolic and diastolic cardiac dysfunction and long-term progression to heart failure^11,12^. The consistency of the constriction is maintained by using a blunt needle of fixed diameter alongside the aorta as a guide. The suture is tied tightly around the needle and the aorta, before the needle is removed, thus creating a permanent constriction. The degree of constriction “tightness” likely dictates the TAC severity and subsequent phenotype progression^13^, since a small diameter needle would produce a more narrow constriction, whereas a larger diameter needle conversely would cause a milder constriction. The most commonly reported needle diameter for TAC is 27 gauge (27G)^5–7,11^, with a minority of studies using 25G^14^, 26G^15^ or 28G^16^. As such, few studies have directly compared the resulting phenotypes of different gauge TAC in great detail^17^. Furthermore, the potentially different effects of varying degrees of pressure overload on LV haemodynamics, as well as on renal structure and function, remain unknown. This is particularly important since the selection of an informative model is critical to successful analysis of genetic or pharmacologic interventions in heart failure.

Here we compared mild (25G), moderate (26G), and severe (27G) TAC model phenotypes and discuss their usefulness as preclinical models in a range of settings. We found a largely graded response to TAC severity in terms of cardiac hypertrophy, dysfunction and fibrosis, representing the transition from compensated to decompensated heart failure phenotypes.

## Results

### Characterization of TAC Surgery

**Figure 1:**
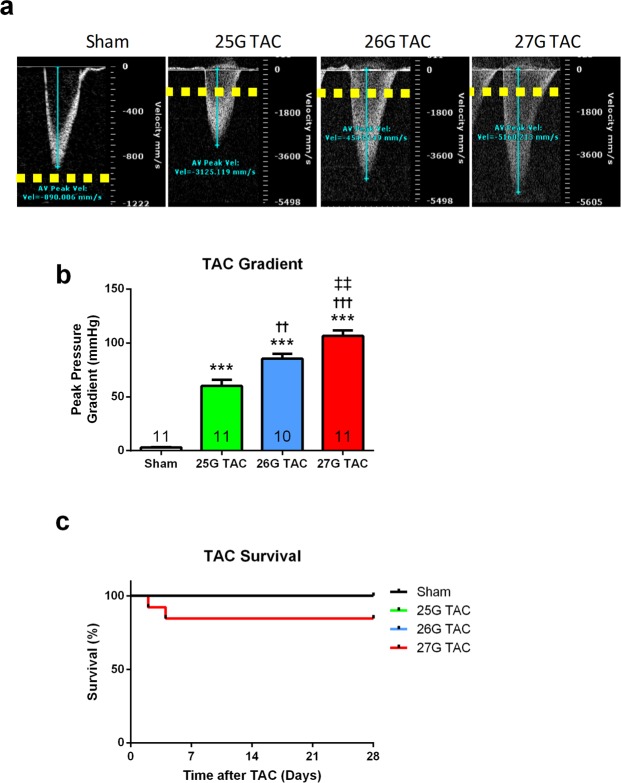
Graded aortic trans-TAC pressure gradients following one week of TAC. (**a**) Representative pulsed-wave Doppler images for sham, 25G, 26G, and 27G TAC mice after 7 days. Yellow dotted line indicates a velocity of 1000 mm/second. (**b**) Peak trans-TAC pressure gradients calculated from Doppler velocities using the Bernoulli equation after 7 days. (**c**) Kaplan-Meier survival curves for 25–27G graded TAC; P > 0.13, log-rank (Mantel-Cox test). ***P < 0.001 vs. sham, ^††^P < 0.01 and ^†††^P < 0.001 vs. 25G TAC, ^‡‡^P < 0.01 vs. 26G TAC by 1-way ANOVA with Tukey’s post-test; n = 10–11.

TAC surgery generated significant, graded echocardiography-derived trans-TAC pressure gradients of 60.2 ± 5.6 mmHg, 85.5 ± 4.4 mmHg and 106.6 ± 5.2 mmHg for 25G, 26G and 27G respectively, compared to 3.0 mmHg for sham mice (P < 0.0001; Fig. 1a and b). Importantly, the trans-TAC gradient was significantly higher in 26G TAC compared to 25G TAC (P < 0.01), and significantly higher in 27G TAC compared to 26G TAC (P < 0.01), representing a distinct, graded phenotype.

Three mice receiving TAC (1 × 26G and 2 × 27G) died either during surgery or within 8 hours of surgery, and hence were not included in the survival analysis. The remaining 25G and 26G TAC mice had 100% survival after the 8-hour perioperative period, whereas 2 of 13 mice (15.4%) subjected to 27G TAC died within 4 days (Fig. 1c; P > 0.05).

### Cardiac Hypertrophy

**Figure 2:**
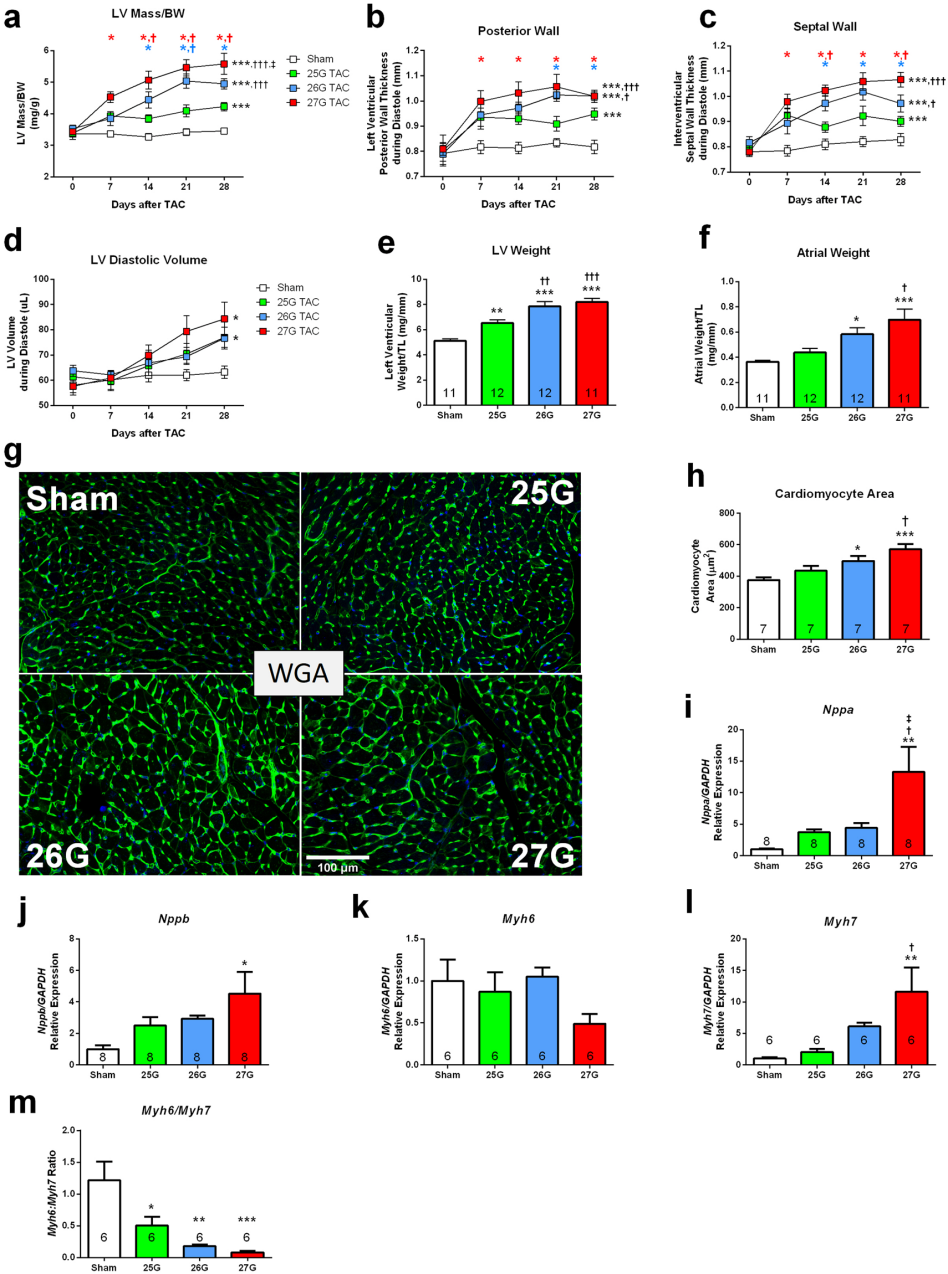
Graded TAC results in graded left ventricular hypertrophy and expression of pro-hypertrophy genes. Echocardiography-derived indices for (**a**) calculated left ventricular (LV) mass normalized to body weight, (**b**) LV posterior wall thickness, (**c**) interventricular septal wall thickness and (**d**) LV volume during diastole. (**e**–**f**) Excised tissue weights normalized to tibia length for left ventricle and left and right atria. (**g**) Representative LV sections stained with wheat germ agglutinin (WGA - green) and DAPI (blue) for assessment of cardiomyocyte area. Scale = 100 µm). (**h**) Quantification of cardiomyocyte area. (**i**–**m**) Quantitative RT-PCR for expression of atrial natriuretic peptide (*Nppa*), brain natriuretic peptide (*Nppb*), Myosin heavy chain-α (*Myh6*) and myosin heavy chain-β (*Myh7*), respectively in apical LV tissue. (**m**) Ratio of *Myh6/Myh7* gene expression. *P < 0.05, **P < 0.01 and ***P < 0.001 vs. sham, ^†^P < 0.05, ^††^P < 0.01 and ^†††^P < 0.001 vs. 25G TAC, ^‡‡^P < 0.01 vs. 26G TAC by 2-way repeated measures ANOVA (**a**–**d**; n = 10–11) or 1-way ANOVA (**e**–**m**; n = 6–8) with Tukey’s post-test. Coloured symbols (**A**–**C**) indicate P < 0.05 vs. sham at the time point indicated.

All mice subjected to TAC developed LV hypertrophy (Fig. 2). Echocardiography detected a graded 22.4%, 43.4% and 61.7% increase in LV mass normalized to body weight, compared to sham for 25G, 26G and 27G TAC mice respectively, after 28 days (Fig. 2a). Mice subjected to 26G TAC had significantly more LV hypertrophy than 25G TAC (P < 0.001), and 27G TAC mice had more than that of 26G TAC (P < 0.05). Further post-hoc analysis showed significant hypertrophy between 27G TAC and sham mice after just 7 days (P < 0.05), whereas this difference emerged after 14 days in mice subjected to 26G TAC. Similar results were obtained when measuring posterior wall thickness (Fig. 2b) and septal wall thickness (Fig. 2c). LV dilation, as measured by calculated LV volume during diastole, increased to a similar extent in all mice subjected to TAC (Fig. 2d).

Cardiac tissue mass at terminal harvest (day 28) supported the echocardiographic data, and showed graded LV hypertrophy in all 3 TAC groups, especially for 26G and 27G TAC mice compared with 25G TAC mice (Fig. 2e; P < 0.01). Significant atrial hypertrophy was absent in 25G TAC, but was present in both 26G and 27G TAC to a similar extent (Fig. 2f; P < 0.05). The response was partially graded, since mice subjected to 27G TAC had significantly more atrial hypertrophy than 25G TAC mice (P < 0.05). The increase in cardiac tissue weight correlated with an increase in individual cardiomyocyte area (Fig. 2g,h). Cardiomyocyte area was significantly increased in 26G TAC (P < 0.05) and 27G TAC mice (P < 0.001) compared to sham. Furthermore, mice subjected to 27G TAC had significantly larger cardiomyocyte areas compared to 25G TAC mice (P < 0.05).

We measured foetal gene expression by qPCR, since re-expression of foetal genes in the myocardium indicates pathological, as opposed to physiological, hypertrophy. We observed an upregulation of the genes for atrial natriuretic peptide (*Nppa*) and brain natriuretic peptide (*Nppb*), in LV apex tissue in mice subjected to 27G TAC, but not in 25G or 26G mice (Fig. 2i,j). Myosin heavy chain (*Myh*) 6 gene expression did not change, but the foetal isoform, *Myh7*, was upregulated 10-fold in 27G TAC mice compared to sham and approximately 2-fold vs. 25G TAC (P < 0.05; Fig. 2k,l). As such, the *Myh6/Myh7* ratio was significantly reduced, but statistically to a similar extent in all TAC mice compared to sham (Fig. 2m; P < 0.05).

Taken together, these results are consistent with graded, pathological cardiac hypertrophy, which is substantially more severe in 27G TAC mice.

### Cardiac Function by Echocardiography

**Figure 3:**
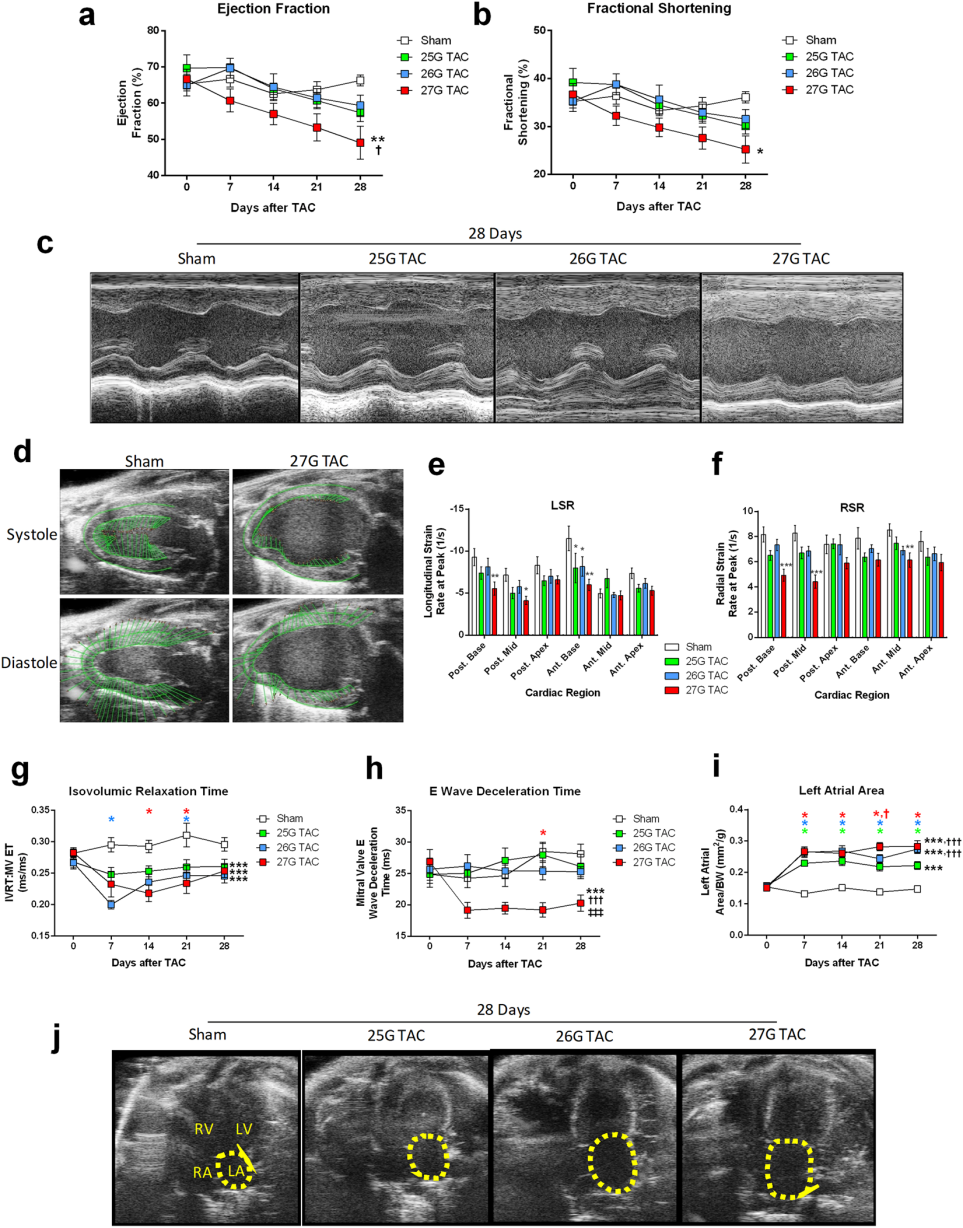
Echocardiography shows graded reduction in cardiac function after 25G, 26G and 27G TAC. (**a**) Left ventricular ejection fraction, (**b**) fractional shortening and (**c**) representative images of M-Mode echocardiography after 28 days show systolic dysfunction. (**d**) Representative images of longitudinal LV strain vector velocities during systole and diastole for sham and 27G TAC mice. The velocity, direction and synchronicity of tissue movement is indicated by the green lines. (**e**) Echocardiography-derived regional LV longitudinal strain rate (LSR) and (**f**) radial SR (RSR) at 28 days after TAC. Diastolic function determined from the apical 4-chamber view, showing (**g**) Isovolumic relaxation time (IVRT) normalized to total ejection time, (**h**) mitral valve E wave deceleration time and (**i**) left atrial area normalized to body weight. (**j**) Representative images of left atrial areas (yellow dotted line) are from 28 days after TAC. *P < 0.05, **P < 0.01 and ***P < 0.001 vs. sham, ^†^P < 0.05 and ^†††^P < 0.001 vs. 25G and ^‡^P < 0.001 vs. 26G TAC by 2-way ANOVA with repeated measures and Bonferroni’s post-test or by simple 2-way ANOVA (**e**–**f**), with Dunnett’s post-test. Coloured symbols indicate *P < 0.05 vs. sham and ^†^P < 0.05 vs. 25G TAC at the time point indicated for the corresponding colour. n = 10–12.

We assessed LV structure, systolic function, and diastolic function by echocardiography pre-operatively and at days 7, 14, 21, and 28 post-TAC. M-Mode analysis in the parasternal long axis detected a significant, progressive decline in systolic function for mice subjected to 27G TAC over 28 days (Fig. 3a–c; P < 0.01). Ejection fraction and fractional shortening were reduced by an average of 17.6% and 11.5% respectively for 27G TAC mice, compared to an increase of 1.01% and 0.8% for sham mice. There was no significant change in these parameters for mice subjected to 25G or 26G TAC.

Speckle-tracking echocardiography provides a more sensitive measure of systolic function than global ejection fraction or fractional shortening^11,18^. We therefore analysed LV strain using speckle-tracking at day 28 post-TAC. Mice subjected to 27G TAC frequently (nine out of eleven; 82%) had impaired displacement properties and subjectively exhibited deformation asymmetry during both contraction and relaxation (Fig. 3d). LV speckle-tracking analysis was used to calculate regional longitudinal strain rate (LSR) and radial strain rate (RSR) in mice 28 days after TAC (Fig. 3e,f). Both LSR and RSR revealed LV dysfunction in mice subjected to 27G TAC, particularly in the basal and mid sections of both the posterior and anterior walls (Fig. 3d–f; P < 0.05). LSR also revealed significant systolic dysfunction in the anterior base region of mice subjected to both 25G and 26G TAC (Fig. 3e; P < 0.05). This suggests early, regional systolic dysfunction, occurring prior to the detection of global systolic impairment in 25G and 26G TAC mice, whilst mice subjected to 27GG TAC already had established, overt dysfunction.

We measured echocardiographic indices of diastolic function and LV filling pressures in the apical 4-chamber view coupled with pulsed wave Doppler imaging. Normalized isovolumic relaxation time (IVRT) was significantly reduced in TAC mice (Fig. 3g; P < 0.05), suggesting increased filling pressures, but not necessarily diastolic dysfunction. By contrast, mitral valve deceleration time, a measure of ventricular stiffness during diastole, was significantly reduced exclusively in 27G TAC mice as early as 7 days after TAC, consistent with increased LV stiffness, a restrictive filling pattern and possible diastolic dysfunction (Fig. 3h; P < 0.001).

Left atrial dilation occurs in response to chronically elevated left ventricular filling pressures, and represents an early marker of diastolic dysfunction^19^. We observed increased left atrial areas in all mice subjected to TAC from 7 days on compared to sham (Fig. 3i,j). The response was graded, with larger left atrial areas measured in 26G and 27G TAC mice compared to 25G.

### LV Pressure-Volume Analysis

**Figure 4:**
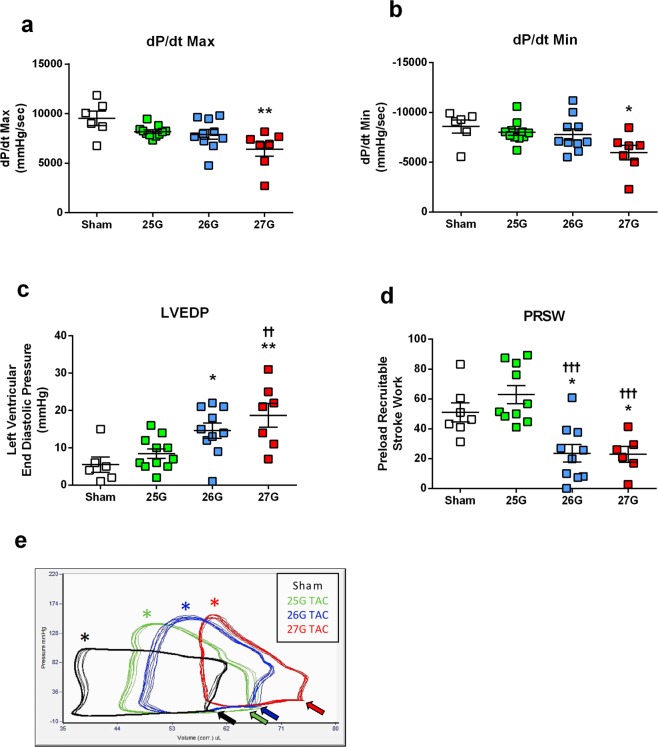
Invasive left ventricular pressure-volume loop analysis shows differential effects on cardiac function after 25G, 26G and 27G TAC. (**a**) Maximal and (**b**) minimal rate of rise in left ventricular pressure (dP/dt) in TAC mice. (**c**) Graded elevation in left ventricular end diastolic pressure (LVEDP). (**d**) Preload recruitable stroke work (PRSW). (**e**) Representative baseline LV pressure volume loops prior to venous occlusion, where coloured asterisks indicate maximum ventricular pressure and arrows denote graded end diastolic pressure. *P < 0.05, **P < 0.01 vs. sham; ^††^P < 0.01, ^†††^P < 0.001 vs. 25G by 1-way ANOVA with Tukey’s post-test; n = 6–11.

Invasive haemodynamic pressure-volume (PV) loop analysis was performed in mice immediately prior to sacrifice, 28 days after TAC. Mice subjected to 27G TAC had reduced haemodynamic cardiac function, with significantly reduced maximal rate of LV pressure rise (dP/dt maximum; P < 0.01) and increased dP/dt minimum (P < 0.05; Fig. 4a,b). Mice receiving 25G and 26G TAC were unchanged. There was a graded elevation of left ventricular end diastolic pressure (LVEDP) in response to TAC severity, rising from 5.5 ± 2.0 mmHg in sham mice to 8.5 ± 1.3 mmHg, 14.6 ± 2.0 mmHg and 18.7 ± 3.2 mmHg for 25G, 26G and 27G TAC respectively (P < 0.01; Fig. 4c,e). Additionally, the LVEDP in 27G TAC was significantly higher than in 25G TAC mice (P < 0.01). Preload-recruitable stroke work (PRSW), a load-independent index of contractile function, was reduced to a similar extent in both 26G and 27G TAC mice, but not in mice subjected to 25G TAC (Fig. 4d).

PV loop-derived functional indices such as cardiac output, stroke volume and stroke work were all reduced in both 26G and 27G TAC mice, but not 25G TAC mice compared to sham (Table 1), independent of heart rate. Stroke work was significantly different between groups (P = 0.02) and interestingly, mice subjected to 25G TAC appeared to have elevated stroke work vs. sham mice (2330 ± 233 uL*mmHg vs. 1767 ± 247 uL*mmHg). End systolic and diastolic pressure-volume relationships (ESPVR and EDPVR), Emax (a contractility index), dP/dt maximum vs. end diastolic volume and effective arterial elastance were all unaffected by surgery. Mice subjected to 25G, 26G and 27G TAC all had elevated aortic pulse pressure compared to sham, which was driven exclusively by an increase in aortic systolic pressure (Table 1). Post-hoc testing revealed that the highest pulse pressures recorded were in 26G TAC mice, which was significantly higher than that of 25G TAC (P < 0.05).

**Table 1:** Left ventricular hemodynamic indices derived from PV loop analysis.

	Sham (n = 6)	25G (n = 10)	26G (n = 10)	27G (n = 7)	P-value
**Pressure-Volume Haemodynamic Measurements (Day 28)**					
Cardiac Output (mL/min)	12.1 ± 1.67	12.8 ± 1.16	8.56 ± 0.87	8.08 ± 1.89	**0**.**0299**
Stroke Volume (µL)	21.5 ± 3.00	23.1 ± 2.01	15.7 ± 1.64	14.7 ± 3.53	**0**.**0415**
Stroke Work (µL*mmHg)	1767 ± 247	2330 ± 233	1510 ± 188	1236 ± 368^†^	**0**.**0229**
Heart Rate (bpm)	558.7 ± 9.33	557.9 ± 12.43	550.2 ± 11.61	551.3 ± 7.026	0.9323
dP/dt Max vs. EDV	181.6 ± 32.9	160.8 ± 55.3	74.13 ± 15.8	49.13 ± 4.62	0.0759
Emax	8.42 ± 1.21	12.8 ± 2.99	8.57 ± 2.10	5.79 ± 0.93	0.2364
ESPVR	2.78 ± 0.43	4.31 ± 0.96	3.18 ± 0.59	2.51 ± 0.17	0.3238
EDPVR	0.30 ± 0.07	0.42 ± 0.12	0.69 ± 0.24	1.03 ± 0.49	0.2613
Effective Arterial Elastance (mmHg/µL)	5.33 ± 1.09	7.00 ± 1.45	12.5 ± 3.17	12.0 ± 2.41	0.113
Aortic Systolic Pressure (mmHg)	102.0 ± 1.71	140.6 ± 6.69**	160.6 ± 7.64***	148.3 ± 3.44***	**<0**.**0001**
Aortic Diastolic Pressure (mmHg)	69.8 ± 1.91	69.5 ± 2.52	66.9 ± 2.71	64.4 ± 1.23	0.4385
Aortic Pulse Pressure (mmHg)	32.2 ± 1.45	71.2 ± 5.35***	93.7 ± 5.84***^,†^	83.9 ± 3.29***	<**0.0001**

EDV: end diastolic volume, ESPVR: end systolic pressure-volume relationship, EDPVR: end diastolic pressure-volume relationship. Data is mean ± SEM. 1-way ANOVA with Tukey’s post-test. Overall ANOVA p-value shown in final column. Post-hoc testing is denoted by *P < 0.05, **P < 0.01 and ***P < 0.001 vs. sham and ^†^P < 0.05 and ^††^P < 0.01 vs. 25G TAC.

### Cardiac Fibrosis

**Figure 5:**
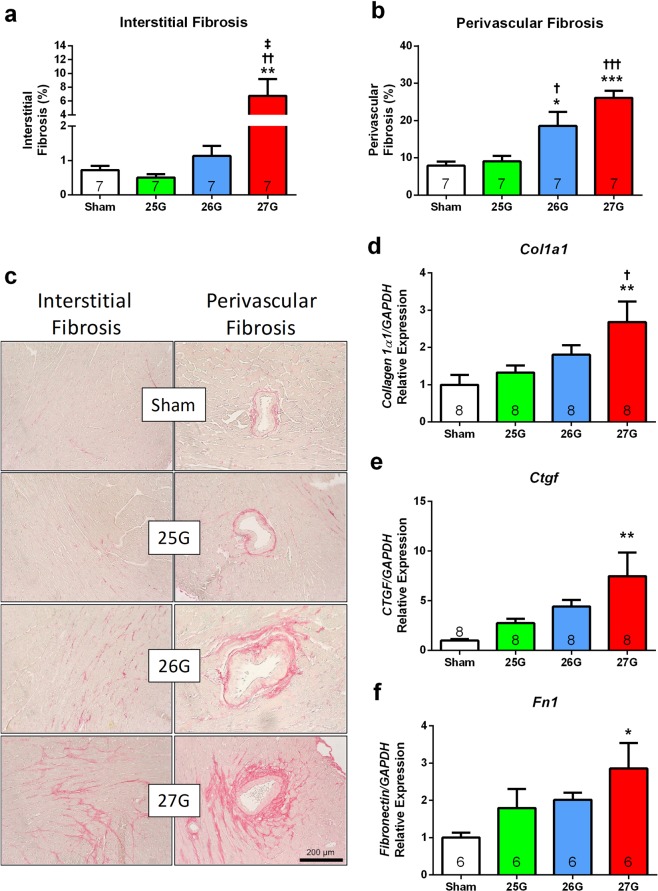
Histological analysis of left ventricular tissue showing graded cardiac fibrosis. (**a**) Quantification of mid left ventricular interstitial and (**b**) perivascular fibrosis with (**c**) representative images. Apical left ventricular gene expression of (**d**) collagen 1a1 (*Col1a1*), (**e**) connective tissue growth factor (*Ctgf*) and (**f**) fibronectin (*Fn1*). *P < 0.05, **P < 0.01 and ***P < 0.001 vs. sham, ^†^P < 0.05, ^††^P < 0.01 and ^†††^P < 0.001 vs. 25G, ^‡^P < 0.05 vs. 26G; 1-way ANOVA with Tukey’s post-test, n = 6–8.

Mice subjected to 27G TAC had marked interstitial and perivascular left ventricular cardiac fibrosis compared to sham control and both 25G and 26G TAC (Fig. 5a–c). In contrast, there was no evidence of interstitial or perivascular fibrosis in mice subjected to 25G TAC (P > 0.98 vs. sham). In mice subjected to 26G TAC, there was also no evidence of interstitial fibrosis, but there was significant perivascular fibrosis (P < 0.05 vs. sham and 25G TAC). Tissue expression of *col1a1*, connective tissue growth factor *(Ctgf)* and fibronectin *(Fn1)* showed upregulation in 27G TAC mice (2.6-fold, 7.5-fold and 2.8-fold vs. sham, respectively; P < 0.001) and a graded, albeit not statistically significant, increase in 25G and 26G TAC mice (Fig. 5d–f).

### Progression to Heart Failure

**Figure 6:**
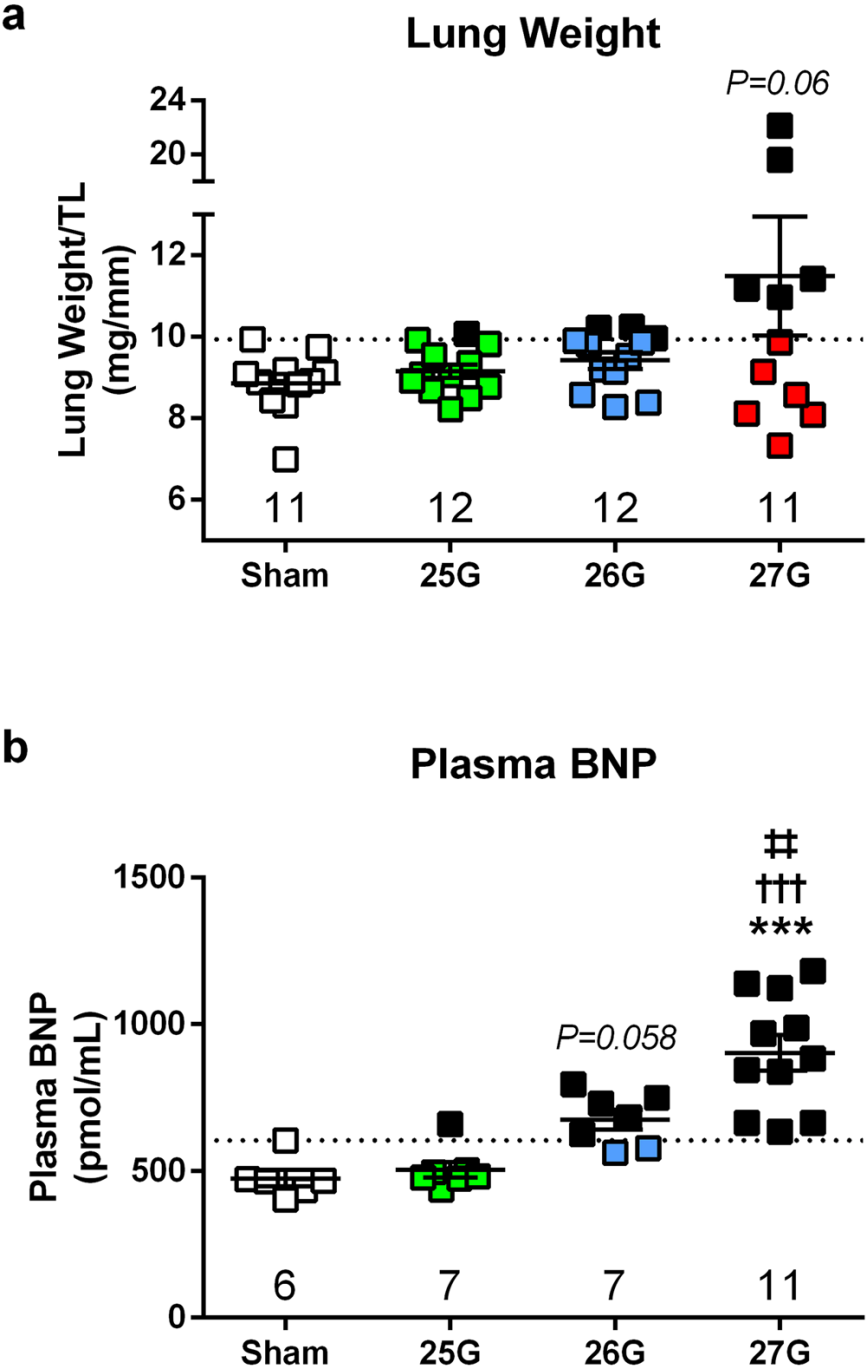
Characterization of TAC-induced heart failure phenotype. (**a**) Lung weight normalized to tibia length measured 28 days after TAC. (**b**) Terminal plasma brain natriuretic peptide (BNP) concentration. Dotted line indicates maximum value for sham mice and black circles indicate a value above that of sham. ***P < 0.001 vs. sham or as indicated, ^†††^P < 0.001 vs. 25G TAC, ^‡‡^P < 0.01 vs. 26G TAC by 1-way ANOVA with Tukey’s post-test; n = 6–12.

TAC produced a variable heart failure phenotype in terms of increased lung weight, as previously described^20^. Lung weight/tibia length ratios were on average 1.3-fold higher in 27G TAC compared to sham, but there was no statistically significant difference (Fig. 6a, P = 0.06). Further analysis revealed that 8% of 25G, 25% of 26G, and 45% of 27G mice developed lung weight/tibia length ratios above that of the maximum value for sham mice, indicating progression to heart failure (black squares; Fig. 6a).

Terminal plasma concentrations of brain natriuretic peptide (BNP), which increases in humans with clinical heart failure, were significantly elevated in 27G TAC mice compared to all others (P < 0.01; Fig. 6b). In contrast, 25G TAC mice did not show any notable elevation in plasma BNP.

### Renal Structure and Function

**Figure 7:**
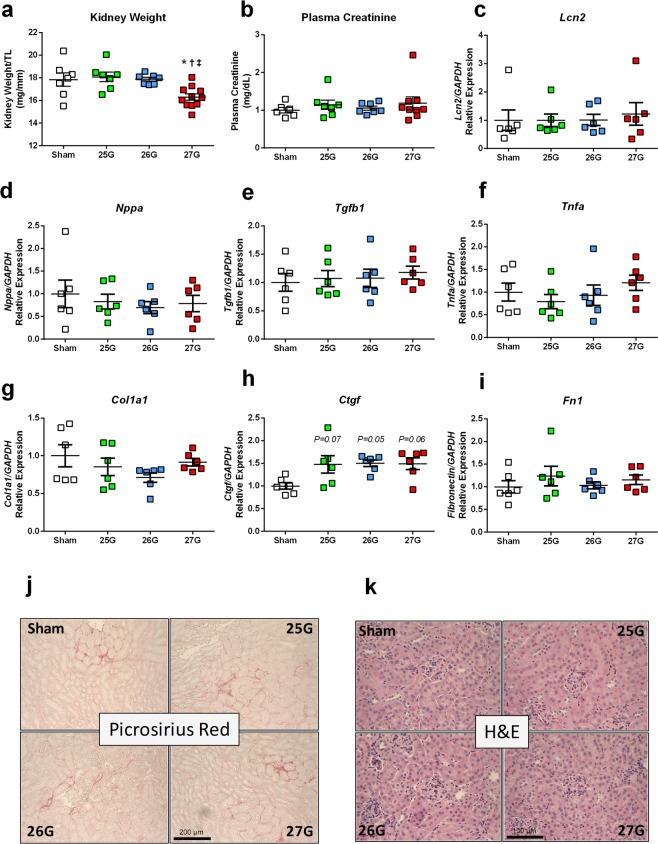
TAC-induced renal changes. (**a**) Terminal kidney weight at 28 days post-TAC. (**b**) Plasma creatinine concentration as an index of renal function at 28 days. (**c**) Renal tissue gene expression for lipocalin-2 (*Lcn2*), (**d**) atrial natriuretic peptide (*Nppa*), (**e**) transforming growth factor β1 (*Tgfb1*), (**f**) tumour necrosis factor-α (*Tnfa*), (**g**) collagen 1a1 (*Col1a1*) and (**h**) connective tissue growth factor (*Ctgf*). (**i**) Representative images for renal fibrosis by picrosirius red staining and (**j**) renal morphology by H&E reveal no overt differences. *P < 0.05 vs. sham, ^†^P < 0.05 vs. 25G, ^‡^P < 0.05 vs. 26G, or P = as indicated vs. sham by 1-way ANOVA with Tukey’s post-test; n = 6–11.

Kidney weights were significantly lower in mice subjected to 27G TAC compared to all other groups when normalized to either body weight or tibia length (Fig. 7a; P < 0.05). We anticipated that this renal atrophy might be indicative of reduced kidney function; however, plasma creatinine, a surrogate for reduced glomerular filtration rate, did not differ between groups (Fig. 7b; P = 0.74). Further, there was no evidence of acute kidney injury in terms of ischaemia or general damage indicated by unaltered tissue neutrophil gelatinase-associated lipocalin-2 (*Lcn2*) gene expression (Fig. 7c).

Renal *Nppa* expression increases in response to a reduction in renal mass, as a compensatory response to maintain sodium homeostasis. The expression of *Nppa* was unchanged in mice subjected to 27G TAC, however, further suggesting that these kidneys did not experience pathological stress (Fig. 7d). Renal expression of transforming growth factor beta-1 (*Tgfb1*) and tumour necrosis factor (*Tnf*) was similar across all groups (Fig. 7e,f respectively); suggesting that weight differences did not represent differential renal inflammation between surgical groups.

TAC surgery did not significantly affect the renal gene expression of *Ctgf*, *col1a1* or *Fn1* (Fig. 7g–i). Importantly, fibrotic gene expression patterns did not differ between TAC groups and thus did not correlate with the renal atrophy observed in the 27G group. Histological staining with picrosirius red showed a similar, basal level of interstitial renal fibrosis in 25G, 26G and 27G TAC groups (Fig. 7j). Finally, haemotoxylin and eosin (H&E) staining of kidney cross sections revealed no overt differences between sham and 25G, 26G or 27G TAC mice in terms of glomerular number, size or morphology (Fig. 7k). Taken together, these results suggest that kidneys of mice subjected to 27G TAC are functionally, structurally and morphologically normal, despite having lower overall mass.

## Discussion

In this study, we induced differing degrees of LV pressure overload in order to directly compare the effects on a wide range of cardiovascular phenotypes. Specifically, we evaluated the effects of mild (25G), moderate (26G) and severe (27G) TAC in mice over 28 days, simultaneously examining organ weights, echocardiography, invasive haemodynamics, gene expression, and renal structure and function. We observed a largely graded response (Table 2). All TAC models had low mortality, significant cardiac hypertrophy, and moderate-severe diastolic dysfunction. Mice subjected to 26G TAC additionally had mild-moderate systolic dysfunction and mild cardiac fibrosis, whereas mice in the 27G TAC group had severe systolic and diastolic dysfunction, severe cardiac fibrosis, and were more likely to exhibit characteristics of heart failure. Based on these findings, we conclude that relatively small differences in TAC constriction produce distinct phenotypes after 28 days.

**Table 2:** Comparison of mild (25G), moderate (26G) and severe (27G) TAC in mice. HFrEF: heart failure with reduced ejection fraction.

Component	25G TAC	26G TAC	27G TAC
Survival	100%	100%	84%
Pressure Overload (TAC Gradient)	60.2 ± 5.6 mmHg (Mild)	85.5 ± 4.4 mmHg (Moderate)	106.6 ± 5.2 mmHg (Severe)
LV Hypertrophy (weight vs. sham)	27.8 ± 4.79% (Mild)	53.5 ± 7.39% (Moderate)	60.4 ± 5.83% (Severe)
Systolic Dysfunction (Echocardiography)	None	Strain only (Mild)	Global and strain (Severe)
Diastolic Dysfunction (Echocardiography)	Moderate	Moderate	Severe
Systolic Dysfunction (PV Loops)	None	Moderate	Severe
Diastolic Dysfunction (PV Loops)	Very mild	Moderate	Severe
Cardiac Fibrosis	None	Perivascular only (Mild)	Perivascular and interstitial (Severe)
Increased Lung Weight (% of total mice)	8% (Very mild)	25% (Mild)	45% (Moderate)
Elevated Plasma BNP (% of total mice)	14% (Mild)	71% (Moderate)	100% (Severe)
Renal Atrophy	None	None	8.65 ± 1.57% (Mild)
Proposed Disease Phenotype Correlate	Hypertensive Heart Disease	Early Decompensation	HFrEF

Since the first report of the TAC model by Rockman and colleagues in 1991^21^, this model has been widely used^11,14,19^, adapted to be less invasive^22–24^, and even combined with other models^25^. In addition to transverse aortic constriction, investigators also have the choice between ascending aortic constriction and abdominal aortic banding^26^, as well as innovative new methods such as the slipknot technique which allows for studies of reversible stenosis, giving insight into cardiac functional recovery and reverse structural remodelling^27,28^. More recently, a study by Melleby and colleagues elegantly described the use of an O-ring for simple, uniform banding of mouse aorta^13^, though it remains to be seen if this method will be widely adopted. Despite these numerous advances, TAC surgery remains the standard pressure overload model. However, the wide phenotypic variability reported with this model^20^, raises the question of whether modest differences in pressure overload can induce predictable differences in pathology. Further, despite the widespread use of TAC, to our knowledge a direct comparison of haemodynamic, echocardiographic, pathologic, and molecular phenotypes between all 3 degrees of pressure overload has not been performed.

From a translational perspective, our cumulative data support that each of the three TAC severities could serve as models for different preclinical phenotypes. We propose that the 25G TAC response at 28 days represents a model of hypertrophy and diastolic dysfunction without systolic dysfunction or cardiac fibrosis, consistent with the human condition of hypertensive heart disease. In the absence of a universally accepted mouse model of heart failure with preserved ejection fraction (HFpEF), this could have high potential for use in drug studies specifically aiming to address diastolic dysfunction/HFpEF in the context of pressure overload. Another potential use would be to investigate a genetic or pharmacological intervention in which an increase in hypertrophy or pathology is expected. Since the 25G TAC promotes a mild hypertrophy phenotype, this would provide a wide window for further exploration.

In contrast, the 26G TAC model represents the transition between compensated and decompensated heart failure, with some systolic dysfunction evident, such as reduced preload-recruitable stroke work and cardiac output in addition to perivascular cardiac fibrosis. The 26G TAC model may therefore be especially useful to study cardiac hypertrophy, dysfunction and fibrosis in genetically modified mice with increased susceptibility to mortality^14,29^. In this situation, cardiac pathology is still observed, but the relative high mortality might be avoided. Conversely, the lack of mortality in WT mice subjected to this stricture might allow greater discrimination to detect heart failure mortality in response to various drug treatments and/or genetic manipulations.

The 27G TAC is the most commonly used model and displays a full range of cardiac pathology. Here, both systolic and diastolic dysfunction were confirmed using sensitive echocardiography and PV loops. We show reductions in global left ventricular ejection fraction and regional strain rate, as well as changes in isovolumic relaxation time, mitral wave E wave deceleration time and left atrial dilation after just 7 days. We also observed marked cardiac fibrosis, hypertrophy and secondary increased lung mass, consistent with pulmonary oedema. These indices are clinically relevant and suggest that this model is extremely useful in both mechanistic studies and preclinical drug studies aiming to ameliorate or reverse the haemodynamic consequences and mortality observed with the heart failure phenotype.

Ventricular function is a key endpoint in preclinical and clinical studies. Here we used a range of techniques to phenotype cardiac function in the various TAC models, finding several important differences. In particular, standard LV M-Mode echocardiography was able to detect systolic cardiac dysfunction in mice subjected to 27G TAC, but not 25G or 26G TAC, whereas PV loops or sensitive LV strain detected more subtle abnormalities in all TAC groups. This is in agreement with previous reports, which suggest the need for more sensitive measures of cardiac function in preclinical studies^11,18,30^. Similarly, diastolic function can also be assessed by a variety of methods, with changes in left atrial area, LVEDP and IVRT regarded as highly sensitive markers of diastolic dysfunction^19,31^. All mice subjected to TAC in the current study had increased left atrial area and decreased IVRT compared to sham; however for mice subjected to 25G TAC, these changes occurred in the absence of increased LVEDP. Left atrial area and IVRT changes may therefore be early markers of elevated filling pressures and diastolic dysfunction in mice, whereas elevated LVEDP occurs with advanced pathology. Taken together, this study reiterates the strengths of using multiple methods to determine systolic and diastolic cardiac function in mice.

From the mechanistic perspective, the finding that different degrees of chronic pressure overload produce unique pathological phenotypes may offer insight into the basic pathophysiology of cardiac remodelling. Multiple prior studies using a single severity of constriction have demonstrated progressive LV hypertrophy which eventually leads to overt LV diastolic and then systolic dysfunction, accompanied by LV chamber dilation and fibrosis. This has led to the prevailing concept that the pathological LV remodelling process represents a single entity that might progress at different rates, or to different degrees, depending on the strength/severity of the pathologic overload^32^. In our study we did in fact observe graded responses in a number of parameters such as LV mass, atrial mass, ratio of *myh*6/*myh*7, and LVEDP. However, other properties such as IVRT worsened to the same degree in all TAC groups, whereas others had more mixed responses that were not graded or step-wise (fractional shortening, ejection fraction, left atrial area, PRSW, perivascular fibrosis). These results support that the cardiac response to various degrees of pressure overload does not simply represent different severities of a single phenotype, albeit with the limitation of the time points evaluated in our study. Rather, our findings suggest a more complex model in which the degree of chronic pressure overload induces differential effects on separate components of the remodelling process. Because different features of LV remodelling predict different outcomes in humans^33^, understanding the molecular and physiological mechanisms governing these selective effects has important clinical implications.

In addition to cardiac phenotypes, we observed renal atrophy in mice subjected to 27G TAC. Renal atrophy was reported in a mouse model of dilated cardiomyopathy, with increased tubulointerstitial and glomerular fibrosis, alongside reduced renal function^34^. Since chronic heart failure can promote chronic kidney failure in both humans and in mouse studies^35^, we decided to investigate this phenotype further and found renal atrophy secondary to 27G TAC, in the absence of renal dysfunction. In addition, we did not find evidence of histological or gene expression changes, including *Lcn2*, a marker and pre-requisite for renal injury and chronic kidney disease^36^. Since long term TAC leads to secondary cardiorenal syndrome, with reduced renal function and increased fibrosis after 12 weeks^37^, we propose the possibility that renal atrophy after 4 weeks observed in the current study may be an early marker of future renal dysfunction. Testing this theory would require further investigation and is beyond the scope of our current study.

In a similar study by Furihata and colleagues, the effects of transverse aortic constriction using needle gauges of 0.400 mm, 0.385 mm and 0.375 mm OD were compared in terms of cardiac structure and function after 28 days^17^. These needle diameters were smaller than the 27G used in the current study, and led to a more accelerated development of heart failure in mice. Whilst their study was the first of its kind and provided insights into gradation of the cardiac hypertrophy response, the smaller needle diameters used are not standard and to our knowledge have yet to be reported elsewhere, possibly owing to the high mortality (up to 52% in 28 days). Furthermore, detailed analysis of cardiac structure and function were lacking beyond wall thicknesses and fractional shortening, making it difficult to comment on distinct phenotypic outcomes.

The current study provides a thorough analysis of the resultant distinct phenotypes; however, our study is not without limitations and considerations. Firstly, all experiments in the current study were performed on male mice and thus we cannot conclude the degree to which these findings apply to female mice. In conducting TAC experiments, it is important to consider the initial size of the transverse aorta, since this will directly influence the degree of constriction. A larger initial aorta, for example in a larger mouse, would lead to a more severe constriction relative to initial size compared to a smaller aorta in a smaller mouse. The current study did not control for initial aortic size; however, mice were age and weight matched with an average starting body weight of 24.9 ± 0.2 g. Promptness of the constriction procedure also has direct consequences on the development of the model. A delay in removing the needle after constriction would lead to prolonged pressure and volume overload in the left ventricle, exposing that mouse to artificially higher initial pathologic stimuli. As such, it is essential to perform this step as quickly as possible for consistency. In the current study we did not directly measure needle removal time, however all TAC surgeries were performed by the same operator, with over 20 years’ experience in the model. Although we performed serial echocardiography, we limited our experimental time point of harvest, haemodynamics, histology, and gene analysis to 28 days after TAC. Accordingly, we cannot determine whether differences in these above variables at 28 days may be magnified or blunted at other experimental time points. However, we chose to focus our analysis on the 28 day time point given the relatively chronic duration of this time period, and its widespread prevalence in other published studies.

In summary, using a standard procedure of minimally invasive TAC, we provide evidence of a graded phenotypic response to increasing degrees of pressure overload, and we conclude that these models produce largely distinct pathological phenotypes, each of which may be useful in different preclinical settings.

## Methods

### In vivo

All experiments were performed in accordance with NIH guidelines, and as approved by the Institutional Animal Care and Use Committee of Tufts University School of Medicine and Tufts Medical Center. Experiments were conducted in accordance with ethical guidelines outlined by Tufts and the NIH. Adult male C57BL/6J mice (24.9G ± 0.2 g and approximately 8 weeks old) were purchased from Jackson Labs (Bar Harbor, ME) and housed in groups of four on a 12 hour light-dark cycle. Mice were kept in a temperature and humidity-controlled room, with free access to food and water.

### TAC Surgery

Mice were randomized to receive minimally invasive TAC surgery with varying degrees of tightness: mild (25G; 0.51 mm outer diameter (OD)), moderate (26G; 0.46 mm OD), or severe (27G; 0.41 mm OD) for 4 weeks, alongside sham-operated controls. TAC surgery was performed with a target of 8 mice per group, based on an expected 80% minimum survival rate from previous studies. All surgical tools were pre-sterilized using a glass bead sterilizer prior to the start of surgery and aseptic technique was used throughout.

For TAC surgery, mice were weighed, then anaesthetised with 2.5% isoflurane gas (in 100% oxygen) in an induction chamber. Mice were transferred to a nose cone and body temperature maintained at 37 °C ± 0.5 °C using a heat pad monitored by rectal thermometer probe. Anaesthesia was maintained throughout at 2.0–2.5% isoflurane in 100% oxygen and mice were confirmed to be at surgical level of anaesthesia by the absence of a toe pinch reflex. Mice were then administered with 1 mg/kg Buprenorphine SR-LAB (ZooPharm, USA) by subcutaneous injection. The left anterior and lateral thorax was then shaved, and the skin prepared for surgery with three povidone iodine/alcohol alternating washes and allowed to dry. The area was then covered with a sterile drape with incision site exposed in centre of the drape. Mice were then intubated using a 22G or 24G angiocatheter and ventilated mechanically (Small Animal Ventilator - Model 687, Harvard Apparatus, USA) at a rate of 80–90 breaths per minute and a tidal volume of 0.2–0.3 mL.

A left thoracotomy was performed by making a 1 cm incision in the mid upper thorax, through which the pectoralis muscles and the intercostal muscles were blunt dissected. The ribs were then retracted using a fine retractor. After retracting the left lung and the thymus, the transverse aorta was identified by its typical location posterior to the thymus gland, and carefully isolated with micro forceps and spring scissors. As quality control, it was essential to carefully dissect away any fat or other tissue structures that would alter the aortic diameter. A 7–0 nylon suture was placed around the transverse aorta (between the brachiocephalic and left common carotid artery) and tied loosely in a single knot. A pre-sterilized, blunt-end 25G, 26G or 27G needle was then placed within the nylon knot, alongside the transverse aorta. Operating quickly, the knot was then tightened fully, encompassing the transverse aorta and the blunt-end needle, and secured with a double knot, before the blunt-end needle was removed. Speed during this step is particularly important, since prolonged occlusion times will artificially and inconsistently increase the effect of left ventricular pressure overload. The incision was closed by suturing in layers. The ribs, intercostal muscles and pectoralis muscles were closed by 6-0 absorbable nylon running sutures. Finally, the skin was closed using 6-0 nylon sutures. Mice were then removed from the ventilator, extubated and anaesthesia stopped. Once sternally recumbent, mice initially recovered under a warming heat lamp and were observed further throughout the perioperative period.

### Echocardiography

Echocardiography was performed as previously described^18,19^, both prior to surgical intervention for baseline cardiac function and again on a weekly basis following TAC surgery. Mice were anaesthetized with 5% isoflurane in medical oxygen at 1 L/minute, then maintained on a nose cone at 1–2% isoflurane on a heated stage in the supine position. Heart and respiration rates were continuously monitored throughout via the stage electrodes. Fur was removed on the chest using depilatory cream (Nair, USA), ensuring that any residual cream was removed fully with water. Ultrasonic gel was applied to the 22–55 MHz echocardiography transducer (MS550D; Vevo 2100, FUJIFILM VisualSonics, Canada) and scanning initiated with parasternal long and short axis views in B-Mode and M-Mode, according to a standard protocol, with a target heart rate of 450–500 bpm.

The apical four-chamber view was used to visualise the mitral valve for indices of diastolic function, including early and late filling velocities (E and A wave), E wave deceleration time, isovolumic relaxation time (IVRT), and total ejection time. The apical four-chamber view was also used to visualise the left atrium for assessment of atrial size.

Following TAC, the peak trans-TAC pressure gradient was determined by application of pulsed-wave Doppler to the aortic arch, distal to the TAC constriction, as previously described^20^. Colour Doppler imaging revealed either laminar blood flow in sham-operated mice, or turbulent blood flow in TAC mice. A 9–18 MHz transducer (MS200) was used to detect higher blood velocities through the TAC constriction using pulsed-wave Doppler. The pulsed-wave peaks were then measured and a peak pressure TAC gradient was calculated using the modified Bernoulli equation (Pressure gradient = 4*velocity^2^)^20^.

All images were analysed using Vevo 2100 software (v3.1.1, FUJIFILM VisualSonics, Canada), taking an average of three measurements per variable per animal. Speckle-tracking echocardiography was used as previously described^19^ using long-axis B-Mode images. Left ventricular longitudinal and radial strain and strain rate were calculated for both epi- and endocardial borders using the semi-automated tracking feature.

### Pressure-volume haemodynamics

On day 28, left ventricular function was determined using *in vivo* pressure volume loop analysis as previously described^38^. Following induction at 2.5% isoflurane, mice were maintained at 2% isoflurane and at 37 °C via a rectal thermometer with automatic feedback to a heat lamp. The right carotid was isolated, clamped, an incision made and a 1.0 F catheter (PVR-1045; Millar Instruments) was advanced through the carotid and into the aortic arch, where aortic pressure was sampled for approximately 30–60 seconds. The catheter was then advanced further and placed inside the left ventricle. The system was calibrated for each animal and pressure and volume recordings measured under basal conditions and with reduced preload by temporarily occluding the inferior vena cava. On occasion, the haemodynamic stress of venous occlusion led to the death of some mice, meaning that occlusion data could not be obtained. Haemodynamic measurements were recorded and analysed with IOX version 2.1.10 software (EMKA Instruments).

### Tissue Isolation

Following pressure-volume haemodynamic measurements, blood was taken directly from the inferior vena cava, heparinised, placed on ice and later centrifuged at 2500 rpm for 15 minutes at 4 °C to obtain plasma, which was stored at −80 °C. The heart was excised, atria removed and the right ventricle separated from the left ventricle. Individual chambers were weighed to assess cardiac hypertrophy. The left ventricle was divided into two pieces: mid-papillary and apical regions. The lungs and kidneys were also weighed and the left tibia length measured for normalization. All tissues were either snap frozen in liquid nitrogen or stored in 10% formalin for histological analysis.

### Assessment and Definition of Heart Failure

TAC mice with lung weight/tibia length ratios above that of the maximum value observed in sham mice were regarded as having a heart failure phenotype, as previously defined^20^.

### Histology

Mid-papillary left ventricular tissue and mid-kidney tissue sections were fixed in 10% formalin for >24 hours and then embedded in paraffin. 5 µm sections were cut, placed onto glass slides, and stained with picrosirius red or H&E. Slides were imaged using a light microscope (Olympus BX40) and SPOT Imaging software (18.2 Color Mosaic; SPOT v5.3, MI, USA) in a blinded fashion at 20x magnification for cardiac tissue and 40x magnification for renal tissue, with ≥3 images per slide. Fibrosis was assessed based on red-orange stain intensity, analysed by ImagePro (Media Cybernetics, MD, USA).

### Gene Expression

Frozen tissue (left ventricular apex or kidney medulla and cortex) was crushed to a fine powder using a cryo-pulverizer at −80 °C and RNA was isolated using a trizol-based extraction kit according to manufacturer’s protocol (Direct-zol™ RNA MiniPrep Plus, Zymo Research, CA, USA). Purified RNA was eluted into 50 µL and RNA concentration was measured using a Nanodrop (Thermo Scientific, USA), followed by reverse transcription of 500 ng RNA for synthesis of cDNA using the iScript cDNA Synthesis Kit (BioRad, USA). Resultant cDNA was diluted with 80 µL water and stored at 4 °C.

Gene expression was performed using the TaqMan™ Gene Expression Assay (Cat# 4331182; Thermo Fisher Scientific, USA). The following probes were used: *Nppa* (Assay ID: Mm01255747_g1), *Nppb* (Assay ID: Mm01255770_g1), *Col1a1* (Assay ID: Mm00801666_g1), *Ctgf* (Assay ID: Mm01192933_g1), *Fn1* (Assay ID: Mm01256744_m1), *Myh6* (Assay ID: Mm00440359_m1), *Myh7* (Assay ID: Mm00600555_m1), *Tgfb1* (Assay ID: Mm01178820_m1), *Tnf* (Assay ID: Mm00443258_m1), *Lcn2* (Assay ID: Mm01324470_m1) and *Gapdh* (Assay ID: Mm99999915_g1) for normalization. Reactions of 20 µL were performed in duplicate (10 µL TaqMan™ Gene Expression Master Mix (Cat# 4369016; Thermo Fisher Scientific, USA), 8 µL water, 1 µL TaqMan™ gene probe and 1 µL cDNA) for 40 cycles of 95 °C for 15 seconds, then 60 °C for 1 minute. Quantitative PCR data was analysed using the delta-delta Ct method, with *Gapdh* as the reference gene. Values were normalised to that of sham mice to represent fold change.

### Plasma BNP

Circulating concentrations of brain natriuretic peptide (BNP) were determined using a BNP ELISA kit (MBS2510603; MyBioSource, USA) according to manufacturer’s instructions. Plasma samples were thawed on ice and diluted 1:1 with assay buffer for a test volume of 100 µL. Test samples were run in duplicate alongside a standard curve of known BNP concentrations on a 96-well plate. Test sample absorbance at a wavelength of 450 nm was compared to the standard curve.

### Plasma Creatinine

An estimate for renal function was determined by measuring plasma creatinine concentration by ELISA (Creatinine Assay Kit (ab65340), Abcam, MA, USA). Plasma was diluted 1:2.5 with assay buffer and run in duplicate alongside a standard curve according to manufacturer’s instructions using the colorimetric detection method. Absorbance at 570 nm was compared to that of the standard curve and converted to mg/dL.

### Statistical analyses

All data is presented as mean ± standard error. The name of the statistical test used is stated in the figure legend alongside replicate (n) numbers. All 2-way ANOVAs with repeated measures were performed using R software (The R Project for Statistical Computing). All other statistical tests were performed using GraphPad Prism v6.0 (La Jolla, CA). A *P-*value of < 0.05 was regarded as statistically significant.

## Data Availability

The datasets generated during and/or analysed during the current study are available from the corresponding author upon reasonable request.
